# Exploring wearable technology use and importance of health monitoring in the hazardous occupations of first responders and professional drivers

**DOI:** 10.1093/joccuh/uiad002

**Published:** 2023-11-09

**Authors:** Sarah Tucker, Soundarya Jonnalagadda, Cheryl Beseler, Aaron Yoder, Ann Fruhling

**Affiliations:** Department of Environmental, Agricultural and Occupational Health, College of Public Health, University of Nebraska Medical Center, Omaha, NE, 68198, United States; Information Systems and Quantitative Analysis, College of Information Science & Technology, University of Nebraska, Omaha, NE, 68182, United States; Department of Environmental, Agricultural and Occupational Health, College of Public Health, University of Nebraska Medical Center, Omaha, NE, 68198, United States; Department of Environmental, Agricultural and Occupational Health, College of Public Health, University of Nebraska Medical Center, Omaha, NE, 68198, United States; School of Interdisciplinary Informatics, College of Information Science & Technology, University of Nebraska, Omaha, NE, 68182, United States

**Keywords:** first responders, professional drivers, perception, wearable, health monitoring, HAZMAT

## Abstract

**Objectives:** Hazardous materials (HAZMAT) pose risks to the health and safety of professionals involved with transportation and emergency responses. Two distinct occupational groups that encounter HAZMAT events are first responders and professional drivers. Wearable technology is a tool that can assist with monitoring the health of professionals involved in HAZMAT events. The aim of this study was to compare and evaluate the perceptions of first responders and professional drivers on wearable technology and attitudes toward health monitoring.

**Methods:** A survey was administered to first responders (*n* = 112) and professional drivers (*n* = 218). Statistical approaches included bivariate analysis, latent class analysis, logistic regression analysis, and path analysis for the variables of interest.

**Results:** There were significant differences between the groups in perceptions of the benefits of monitoring certain health indicators. Professional drivers were more likely to have a history of wearable technology use compared with first responders (odds ratio [OR] = 10.1; 95% CI, 4.42-22.9), reported greater exposure to HAZMAT (OR = 4.32; 95% CI, 2.24-8.32), and were more willing to have their health data monitored by someone other than themselves (OR = 9.27; 95% CI, 3.67-23.4). A multinomial regression model revealed that occupation was not a significant predictor of class preference for acceptance of monitoring specific health indicators.

**Conclusions:** Occupation appeared to be important but further analysis uncovered that characteristics of individuals within the occupations were more salient to the use of wearable technology. HAZMAT exposure, someone else monitoring health data, and experience with wearable technology use were found to be important factors for perceptions about benefits of health monitoring with wearable technology.

## Teaser

**Key points:**

- Wearable technology can be useful to protect health and safety of high-risk occupations. Before integrating real-time monitoring of occupational groups, perceptions and acceptability of wearable technology must be assessed. The perceptions of wearable technology for real-time monitoring differed between characteristics of individuals within occupations rather than the occupation itself. Key variables identified in this study can guide integration of wearable technology to protect the health and safety of high-risk occupations potentially exposed to HAZMAT.

## 1. Introduction

First responders such as firefighters, paramedics, and police officers face significant dangers, injuries, and fatalities in their line of work.^1^ They are at risk of exposure to chemicals, drugs, and other hazardous substances, leading to adverse acute or chronic health effects. Fatigue is a common issue among first responders due to physical exhaustion, cognitive task, sleep cycles, burnout, and emotional stress.^2^ Firefighters are at high risk of injury on the fireground, with traumatic injuries, cuts, bruises, burns, asphyxiation, and respiratory injuries being the most common.^3^ Physical stress, being lost or trapped in a fire situation, and vehicle crashes are the primary causes of death.^3^ Carbon monoxide poisoning is a danger at every fire,^4^ with half of on-duty firefighter deaths being attributed to heart attacks or stroke.^3^ Psychological challenges also affect first responders, including exposure to traumatic events, prolonged exposure to stress, and the potential for mental health conditions.^5^^,^^6^ Overall, the nature of the work of first responders presents a significant risk to their physical and mental health, and there is a need for measures to protect them from these dangers.

Professional drivers who transport hazardous materials (HAZMAT) face multiple hazards in their work,^7^ including extended sitting, whole-body vibration, uncomfortable posture, and repeated motions, which can lead to musculoskeletal pain.^8^ They also have poor cardiometabolic risk profiles,^9^ including overweight and obesity,^10^ hypertension, hypercholesterolemia, high blood glucose, poor mental health, and cigarette smoking.^9^ Transporting HAZMAT increases the risk of incapacitation or fatal injury experienced by professional drivers in single- and multi-vehicle crashes.^11^^,^^12^ Short sleep durations have been associated with motor vehicle accidents, with fatigue also an issue for driver alertness and performance.^13^ HAZMAT spills from truck crashes^14^ can cause significant casualties, injuries, and damage to the environment and property. Therefore, designing solutions to maintain driver alertness and lessen the number of slips, trips, and falls is crucial.

A variety of chronic health concerns threaten the well-being of firefighters and professional drivers, with cancer and coronary heart disease being among the most common.^15^ The inhalation of toxic chemicals from smoke, as well as exposure through the skin, puts firefighters at a higher risk of developing cancer.^16^^,^^17^ In addition, they may suffer from respiratory system problems^18^^,^^19^ when working without respiratory protection during overhaul. Prolonged exposure to extreme physical, mental, and psychological demands, as well as cardiovascular strain and altered circadian rhythm, can lead to body stressors that contribute to coronary heart disease.^20^ Being overweight or obese,^21^^,^^22^ suffering from hyperthermia, and having a sedentary lifestyle also increase the risk of coronary heart disease,^23^ which is the leading cause of death for first responders. Professional drivers, on the other hand, face health threats such as sitting for prolonged hours, exposure to vibration, and physical fatigue.^24^ They have significantly higher body mass index, current cigarette use, and low physical activity compared with US drivers overall.^25^ The length of exposure, toxicity, routes of exposure, and individual health and physical conditions all play a role in the severity of the health effects.

Wearable health devices are increasingly being used by first responders and transportation workers to monitor their body’s reactions in hazardous conditions, manage their efforts, and assess their occupational health.^26^^,^^27^ These devices can provide real-time feedback on an individual’s vital signs, enabling them to assess their occupational health and take necessary actions in emergency situations.^28^ These wearables have the potential to aid cardiologists in studying stress and fatigue among first responder professionals.^29^ Wearables can also provide real-time feedback and interventions to manage cognitive fatigue and prompt breaks or adjust environmental factors to reduce cognitive load.^30^^,^^31^ Devices can detect proximity to hazardous locations and moving equipment, provide early warnings for potential exposure to HAZMAT or environmental conditions, remotely monitor lone workers, and provide timely emergency response or evacuation notifications.^32^ Although drivers are aware of their unhealthy lifestyle, they have concerns regarding continuous monitoring by their employers.^33^ However, in order to avoid accidents, supervisors must ensure that workers are instantly informed of possible hazards. Wearables can be used to notify their superiors about their location, fatigue levels, health condition, and the surrounding environment.^27^ Continuously tracking health physiological changes may lead to early diagnosis of disease and initial treatment, improving their quality of life.^34^ The measurement of multiple signals, such as blood pressure, heart rate, respiratory rate, and blood oxygen saturation levels, can bring greater insight into the pathophysiology of disease and new indications of physiological markers of disease status.^35^

The significant risks associated with HAZMAT highlight the importance of monitoring and mitigating potential hazards to protect the safety of first responders, professional drivers, and the general public. Although wearable technology offers better insights and monitoring schemes, usability is still a concern. Wearable technology must be easy to use for the wearer.^31^ There is limited research exploring first responders’ and professional drivers’ current use of wearable health monitoring technology, which health indicators are perceived as important to them, and/or who should monitor their information during emergencies.

### 1.1. Aims

This study is part of a larger project that involves developing continuous health and environment monitoring systems for professional drivers and first responders who are exposed to HAZMAT. The REaCH (Real-Time Emergency Communication System for HAZMAT Incidents) application was created for real-time monitoring of workers with wearable devices to capture individual health indicators and environmental exposures.^36^ In this specific study the focus was on exploring the health indicators.

Before the REaCH application can be implemented, more information is required for health indicators to be monitored, sensors to be used, and acceptability of continuous monitoring by the users as well as barriers to use. The questions were: (1) Do first responders and professional drivers differ in their history of using wearable technology? (2) Do first responders and professional drivers differ in their views of who should monitor their health data collected using wearable technology? (3) What health indicators do first responders and professional drivers consider useful to monitor in the work field? (4) Are there patterns in the acceptance of monitoring specific health indicators in the sample of first responders and professional drivers? (5) What characteristics, such as a history of using wearable technology, exposure to HAZMAT, or views on who should monitor health data, explain any patterns identified in the acceptance of measuring health indicators? (6) What are the barriers for first responders and professional drivers using wearable technology?

The objective of this study was to compare and evaluate the perceptions of first responders and professional drivers on using wearable technology and attitudes towards health monitoring.

## 2. Methods

### 2.1. Survey preparation

A survey was prepared to identify feasibility of first responders using wearable technology, current use of health monitoring technology, and perception of health monitoring. The survey was administered in 2019 by the College of Public Health at the University of Nebraska Medical Center on behalf of the REaCH project.^37^ The survey was sent to 24 local area fire departments and collected by first responders’ supervisors. There were no inclusion/exclusion criteria. The survey was approved by the Institutional Review Board (IRB) (IRB# 691–17-EX) of the University of Nebraska Medical Center. A similar survey was prepared for professional drivers in 2022, which included demographics and perception of wearable technology.^38^ The survey was reviewed by 3 experts in the trucking industry before it was administered to ensure questions were clear and terminology was suitable for professional drivers. The experts included a HAZMAT professional from the local state patrol, a Safety Manager for a HAZMAT trucking company, and a Commercial Driver’s License (CDL) Truck Driver Instructor. The professional driver survey was distributed electronically through multiple sources including trucking industry companies, professional organizations, and Facebook Ads. Facebook Ads were the most sufficient strategy in obtaining respondents due to barriers encountered during the COVID-19 pandemic. Employers were hesitant or declined to distribute a health-related survey. The surveys are included in Appendix A.

### 2.2. Statistical approaches

The total sample comprised 115 first responders and 218 professional drivers for a total of 333 participants. The final sample with complete data was 261. The chi-squared test for independence was used to compare acceptance of measuring health indicators using wearable technology between the 2 groups. Characteristics that might influence acceptance of perceptions were compared using the chi-squared test. Latent class analysis (LCA) was used to capture person-centered effect of the sample to identify subgroups of individuals based on heterogeneity in preferences for monitoring health indicators. The probabilities were used to classify respondents into a specific subgroup or class. The patterns of responses were then tested to assess characteristics that might explain the subgroups. Logistic regression analysis was conducted to examine the effect size for occupational groups and ever or currently using wearable technology, exposure to HAZMAT, and who should monitor health indicators. A multinomial generalized linear model was used to test whether occupational group and other covariates were associated with the class assignments from the LCA. The model was built by adding the occupational group first as it was the primary variable of interest, followed by ever or currently using wearable technology, exposure to HAZMAT, and who should monitor health data. Based on the results from individual regression models, path analysis was used to build a proposed conceptual model of how the characteristics fit together to explain the use of wearable technology. The statistical analyses were conducted in R software. More detailed methods can be found in Appendix B.

## 3. Results

The results from bivariate analysis, shown in Table 1, compared the first responders’ and professional drivers’ wearable technology use, exposure to HAZMAT at work, views on who should monitor health data, and specific health indicators using wearable technology indicated as useful to monitor (yes/no). The bold values indicate statistically significant results at *P* < .05. First responders and professional drivers differed significantly in ever use of wearable technology (*P* < .0001), current use of wearable technology (*P* < .0001), exposure to HAZMAT (*P* < .0001), and who should monitor health data (*P*< .0001). The specific health indicators that first responders and professional drivers identified as useful to monitor (yes/no) that significantly differed were heart rate (*P* = .004), blood pressure (*P* < .0001), core body temperature (*P* < .0001), stability (maintenance of body position) (*P* < .0001), blood oxygen levels (*P* = .003), respiration carbon dioxide levels (*P* = .0004), and cortisol levels (*P* < .0001).

**Table 1:** Characteristics of first responders (*n* = 112) and professional drivers (*n* = 159) and their preferences for monitoring health indicators using wearable technology.

**Variable**	**First responders *n* (%)**	**Professional drivers** ***n* (%)**	**Chi-squared** **(*P* value)** ^**a**^
**Ever used wearable technology** **Yes** **No**	46 (45.1) 56 (54.9)	142 (89.3) 17 (10.7)	**60.3** **(<.0001)**
**Currently use wearable technology** **Yes** **No**	28 (27.5) 74 (72.5)	134 (84.3) 25 (15.7)	**85.2** **(<.0001)**
**Exposed to hazardous materials at work** **Yes** **No**	31 (30.7) 70 (69.3)	117 (75.0) 39 (25.0)	**49.3** **(<.0001)**
**Preferred person to monitor health data** **No one** **Myself** **Someone else** **Myself and someone else**	2 (2.0) 25 (25.0) 10 (10.0) 63 (63.0)	2 (1.26) 34 (21.4) 67 (42.1) 56 (35.2)	**32.2** **(<.0001)**
**Barriers to using wearable technology in those who are not currently using it** **Cost** **Lack of interest** **Durability** **Do not own one** **Lack of accuracy** **Privacy**	16 (21.0) 38 (50.0) 8 (10.5) 12 (15.8) 1 (1.3) 1 (1.3)	0 14 (60.9) 0 9 (39.1) 0 0	N/A
**Heart rate** **Yes** **No**	100 (98.0) 12 (2.0)	140 (88.1) 19 (11.9)	**8.38** **(.004)**
**Blood pressure** **Yes** **No**	95 (93.1) 7 (6.9)	110 (69.2) 49 (30.8)	**21.2** **(<.0001)**
**Core body temperature** **Yes** **No**	91 (89.2) 11 (10.8)	101 (63.5) 58 (36.5)	**21.1** **(<.0001)**
**Skin temperature** **Yes** **No**	68 (66.7) 34 (33.3)	98 (61.6) 61 (38.4)	0.68 (.41)
**Hydration level** **Yes** **No**	88 (86.3) 14 (13.7)	124 (78.0) 35 (22.0)	2.80 (.09)
**Stability (maintenance of body position)** **Yes** **No**	48 (47.1) 54 (52.9)	141 (88.7) 18 (11.3)	**53.9** **(<.0001)**
**Falls** **Yes** **No**	52 (51.0) 50 (49.0)	91 (57.2) 68 (42.8)	0.98 (.32)
**Breathing rate** **Yes** **No**	89 (87.3) 13 (12.7)	120 (75.5) 39 (24.5)	5.41 (.02)
**Breathing depth** **Yes** **No**	65 (63.7) 37 (36.3)	107 (67.3) 52 (32.7)	0.35 (.55)
**Blood oxygen levels** **Yes** **No**	93 (91.2) 9 (8.8)	122 (76.7) 37 (23.3)	**8.93** **(.003)**
**Respiration CO**_**2**_ **levels** **Yes** **No**	87 (85.3) 15 (14.7)	104 (65.4) 55 (34.6)	**12.5** **(.0004)**
**Cortisol levels (stress)** **Yes** **No**	88 (86.3) 14 (13.7)	103 (64.8) 56 (35.2)	**14.6** **(<.0001)**
**Skin resistance (stress and hydration)** **Yes** **No**	72 (70.6) 30 (29.4)	113 (71.1) 46 (28.9)	0.007 (.93)
**Classes of 13 health measures** **High preference for measuring health** **Medium preference for measuring health** **Low preference for measuring health**	2 (2.0) 49 (48.0) 51 (50.0)	15 (9.43) 83 (52.2) 61 (38.4)	**7.50** **(.02)**

a Bold values indicate statistically significant results at *P*<.05.

Abbreviation: N/A, not available.

**Figure 1:**
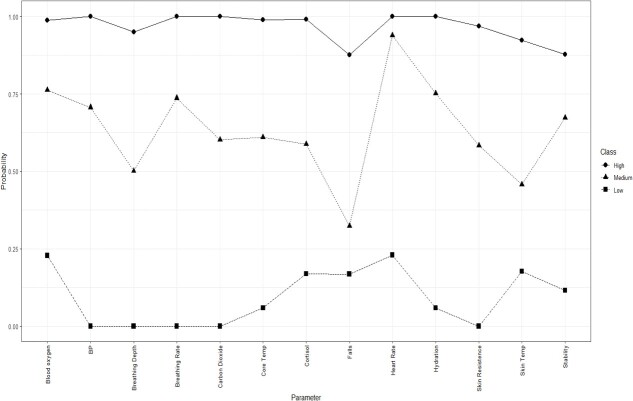
Estimated probability of class inclusion for 13 possible health indicators.

Table 2 shows the model fit for the LCA, which identified 3 classes (low, medium, and high preference). Plotting this 3-class solution demonstrated that it was theoretically sound because it was based on varying frequency of acceptance of the health indicators on the *x*-axis (Figure 1). The lines of the 3 groups did not cross, which revealed 3 distinct levels of preference of health monitoring. The subgroup that strongly accepted the monitoring of nearly all health indicators was only 6.5% of the sample (*n* = 17), whereas the largest subgroup (51%) was those in the medium category. Fifteen professional drivers were in the subgroup of 17 respondents who strongly favored monitoring health indicators (binomial probability of 0.001). Table 3 (Kruskal-Wallis chi-squared used due to small cell sizes) shows the distribution of characteristics in the LCA, which included ever use of wearable technology, current use of wearable technology, exposure to HAZMAT, and who should monitor health data.

**Table 2:** Model fit parameters for 2 to 4 classes of preference for measuring health indicators using wearable technology in 261 first responders and professional drivers.^a^

**Model fit statistic**	**1-class solution**	**2-class solution**	**3-class solution**	**4-class solution**
**AIC**	3767	3215	3049	3012
**BIC**	3813	3311	**3195**	3208
**Entropy**	N/A	0.906	**0.927**	0.882
**Smallest class count**	N/A	0.471	0.065	0.065
**Smallest class size**	N/A	123	17	17

a Shown in bold are low BIC value and high entropy, which indicates best model fit.

Abbreviations:AIC, Akaike information criterion; BIC, Bayesian information criterion; N/A, not available.

**Table 3:** Distribution of wearable technology use, exposure to hazardous materials, and preferences for monitoring by class.

**Characteristic**	**Low preference class** ***n* = 112 (42.9%)**	**Medium preference class** ***n* = 132 (50.6%)**	**High preference class** ***n* = 17 (6.51%)**	**Chi-squared** **(*P* value)** ^**a**^
**Ever use wearable technology** **Yes** **No**	13 (23.5) 4 (23.5)	74 (66.1) 38 (33.9)	101 (76.5) 31 (23.5)	3.46 (.18)
**Currently use wearable technology** **Yes** **No**	13 (23.5) 4 (23.5)	64 (57.1) 48 (42.9)	85 (64.4) 47 (35.6)	2.96 (.23)
**Exposed to hazardous materials** **Yes** **No**	14 (82.3) 3 (17.7)	51 (46.8) 58 (53.2)	83 (63.4) 48 (36.6)	**11.3** **(.004)**
**Preference for health data monitoring** **No one** **Myself** **Others** **Both**	0 0 12 (75.0) 4 (25.0)	2 (1.80) 35 (31.5) 25 (22.5) 49 (44.1)	2 (1.52) 24 (18.2) 40 (30.3) 66 (50.0)	2.88^b^ (.24)

a Bold values indicate statistically significant results at *P*<.05.

b Kruskal-Wallis chi-square used due to small cell sizes.

Multivariable analysis revealed that professional drivers were more likely to ever use wearable technology (odds ratio [OR] = 10.1; 95% CI, 4.42-22.9), more likely to be exposed to HAZMAT (OR = 4.32; 95% CI, 2.24-8.32), and to think that someone other than themselves could monitor their health data (OR = 9.27; 95% CI, 3.67-23.4). Replacing ever using wearable technology with currently using wearable technology produced similar results, although with a higher OR for current use (OR = 14.7; 95% CI, 6.84-31.6). Exposure to HAZMAT (OR = 3.91; 95% CI, 1.95-7.84) and others monitoring health data (OR = 10.2; 95% CI, 3.96-26.4) showed only slight changes. Each of the variables significant in the chi-squared analysis remained significant and improved model fit when included in the model of occupational group. The differences between first responders and professional drivers were in past or current use of wearable technology, whether someone else should monitor health data, and exposure to HAZMAT. Professional drivers were more likely to accept all 3 compared with first responders. Exposure to HAZMAT significantly increased the odds of accepting that others could monitor personal health data in an unadjusted model (OR = 2.02; 95% CI, 1.14-3.58).

In the multinomial regression model with the 3 classes as the outcome variable, the medium preference group was compared with the low preference and the high preference groups. After adding exposure to HAZMAT and having others monitor health indicators in a hierarchical approach, occupation was no longer significant in the model (Table 4). The likelihood ratio test indicated that Model 4 was statistically identical to Model 5; these 2 models fit the data equivalently. The small size of the high preference group, which included at most 17 respondents, resulted in wide CIs, although with large ORs. HAZMAT remained significant in the model in the 131 who accepted monitoring at low levels.

**Table 4:** Multinomial regression analysis with subgroup (class) as the outcome in 255 first responders and professional drivers with the medium group as the reference category.

**Variable**	**High preference class** **OR (95% CI)**	**Low preference class** **OR (95% CI)**
**Model 1:**	*n* = 17	*n* = 132
**Professional drivers vs first responders**	6.27 (1.37-28.7)	1.42 (0.85-2.37)
**Model 2:** **Professional drivers vs first responders** **Exposure to hazardous material**	*n* = 16 3.89 (0.78-19.4) 3.27 (0.83-13.0)	*n* = 131 1.08 (0.60-1.91) 1.91 (1.08-3.38)
**Model 3:** **Professional drivers vs first responders** **Exposure to hazardous material** **Preferring others to monitor health data**	*n* = 16 3.35 (0.37-30.2) 4.74 (0.94-23.8) 7.01 (1.97-25.0)	*n* = 131 0.95 (0.51-1.73) 1.99 (1.12-3.54) 1.39 (0.74-2.60)
**Model 4:** **Exposure to hazardous material** **Preferring others to monitor health data**	*n* = 16 6.32 (1.33-30.0) 8.72 (2.53-30.1)	*n* = 131 1.95 (1.15-3.28) 1.37 (0.75-2.49)

Abbreviation: OR, odds ratio.

**Figure 2:**
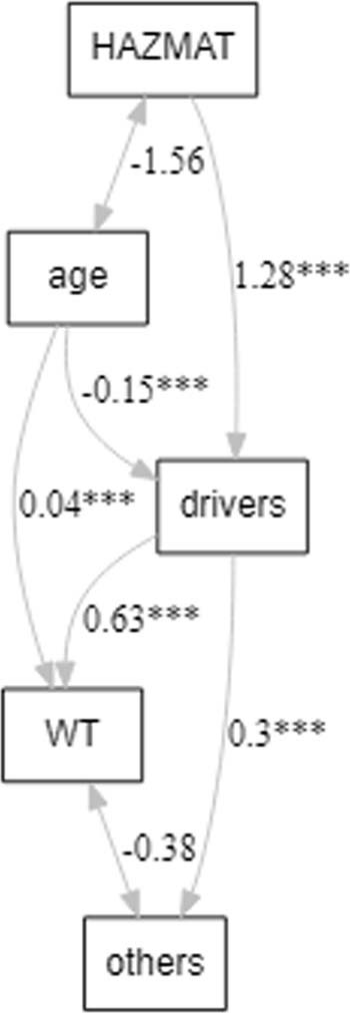
Path analysis showing that being a professional driver mediates the relationship between exposure to hazards and preferring that others monitor personal health data.

Path analysis was used to better understand the order of associations among the variables shown in Table 4. Exposure to HAZMAT was positively and significantly associated with being a professional driver (driver), which was positively associated with currently using wearable technology (WT) and preferring others to monitor health data (others) (Figure 2). Age was negatively correlated with being exposed to HAZMAT and positively associated with using wearable technology. The chi-squared statistics were not significant (*P* = .68), the comparative fit index and Tucker-Lewis index were 1.00, and the root mean square error of approximation was 0. Reversing the order of variables in the path model did not fit the data, suggesting the path shown in Figure 2 is the most probable fit to the data.

There were not enough responses (*n* = 23 for professional drivers and *n* = 76 for first responders) to statistically analyze the response to barriers of using wearable technology. Barriers reported among the professional drivers were lack of interest and not owning a wearable device. Of the 76 first responders who responded, 50% reported lack of interest, 21% reported cost, 16% reported not owning a device, and 11% reported durability.

## 4. Discussion

Initial results showed that compared with first responders, professional drivers were more likely to ever use wearable technology, more likely to be exposed to HAZMAT, and to think that someone other than themselves could monitor their health data. Past use of wearable devices has been shown to positively influence willingness to use wearable technology in the occupational setting. The perception of benefits from wearable technology has also been shown to influence attitude toward using wearable technology.^39^ Even though both groups in this study were different occupations, it was appropriate to combine them into 1 class analysis because both groups had similar potential exposure to HAZMAT. Also, first responders and professional drivers require routine physical exams.

### 4.1. History of using wearable technology

Both bivariate analysis and multivariable analysis showed that compared with first responders, professional drivers were more likely to ever use wearable technology, which was statistically significant. This was an important finding because the history of wearable devices can influence willingness to use wearable technology at work. A study by Häikiö et al^40^ showed that construction workers with experience of wearable devices positively influenced willingness and interest of using wearable technology during the workday. Although construction workers were a different occupational group, they had similar potential exposure to HAZMAT, physical demands, and weather extremes, to occupations in this study. The history of using wearable technology differed between first responders and professional drivers.

### 4.2. Views on monitoring health data

The views of who should monitor health data from wearable technology were evaluated between first responders and professional drivers. Compared with first responders, professional drivers were more agreeable that someone other than themselves could monitor their health data. No statistical interactions were seen between the first responders and professional drivers. Professional drivers may be more comfortable being monitored on an individual level, which might be another factor for further exploration. First responders were more commonly monitored on a group level through the air packs with less individual monitoring.

### 4.3. Useful health indicators

The perceived usefulness of health indicators to monitor in the work field was evaluated between responders and professional drivers. First responders and professional drivers differed in their responses to what health indicators should be monitored. The question was asked, “In your opinion, what types of health information would be useful to monitor for [first responder or professional driver] safety when working in the field?” In Table 1, first responders rated monitoring the following health indicators as more acceptable (meaning a greater percentage of first responders answered “yes”) compared with professional drivers: heart rate, blood pressure, core body temperature, blood oxygen levels, respiratory carbon dioxide levels, and cortisol levels. Professional drivers rated stability as more acceptable compared with first responders. First responders may be more familiar with health indicators and terminology compared with professional drivers. With background medical knowledge, first responders might be more accepting of monitoring certain health indicators like vital signs and rated these as more acceptable. Most of the vital signs except for breathing rate and depth were rated as more acceptable by a greater percentage of the first responders than professional drivers. This result was consistent with data previously collected from the focus group session with first responders from a local HAZMAT response team. Short- and long-term health and safety were the majority of concerns reported by first responders in the focus group.^36^

Next, patterns in the acceptance of monitoring specific health indicators in the sample of first responders and professional drivers emerged. The LCA revealed 3 class preferences for acceptance of monitoring of 13 possible health indicators: low, medium, and high preference classes. The low preference class was the group that weakly accepted monitoring of health indicators, and the high preference class was the group that strongly accepted monitoring of almost all health indicators. The medium preference class was the reference group. Fifteen out of the 17 individuals in the high preference class were professional drivers whereas most first responders fell into the low and medium classes. Characteristics of professional drivers indicated that professional drivers were more frequently exposed to HAZMAT compared with first responders. When exposure to HAZMAT and having others monitor health data indicators were added to the multinomial regression model, occupation was no longer significant. This suggested that not occupation, but rather characteristics (past wearable technology use, exposure to HAZMAT, and allowing others to monitor health data) of the first responders and professional drivers were factors in class membership in those who strongly accepted health monitoring of most of the health indicators. Professional drivers might perceive greater exposure potential; therefore, they may be more open to the idea of their health data being monitored. These results suggest that the perception of being exposed to HAZMAT and being amenable to having others monitor their health data partially explain class membership. The users of wearable technology would need to understand the value of health monitoring in order to move from a low to medium or medium to high preference class.

### 4.4. Acceptance of measuring health indicators

Characteristics such as history of using wearable technology, exposure to HAZMAT, or views on who should monitor health data explained patterns identified in the acceptance of measuring health indicators. The results indicated that patterns in acceptance of measuring health indicators were not related to occupation. The characteristics of the groups were more predictive for class rather than being a first responder or a professional driver. These characteristics included age, exposure to HAZMAT, use of wearable technology, and allowing others to monitor health data. The order of associations in the path analysis revealed that exposure to HAZMAT was positively and significantly associated with being a professional driver (driver), which was positively associated with using wearable technology (WT) and preferring others to monitor health data (others) (Figure 2). Age was negatively correlated with being exposed to HAZMAT and positively associated with using wearable technology. A greater perceived risk of HAZMAT exposure might lead to perceived greater benefit of health monitoring, which might explain greater acceptance of having someone else monitor health data. The path analysis showed that being a professional driver mediated the relationship between exposure to HAZMAT and preferring that others monitor personal health data.

### 4.5. Barriers to using wearable technology

Several barriers resulted from the sample of respondents. Although some barriers to use differed between first responders and professional drivers, both groups reported a lack of interest as a barrier. The reported lack of interest in using wearable technology of both groups could be attributed to lack of clear individual benefit. Another barrier professional drivers reported was not owning a wearable device, which could be linked to lack of interest. First responders reported cost and durability as barriers. Cost could be related to the upfront cost of purchasing wearable technology or replacement costs. Durability could be attributed to the working conditions. Their concern for durability of wearable technology was consistent with the type of environment they might encounter, for example, extreme heat. Professional drivers work in a more controlled environment and may not be as concerned with durability. Cost was a barrier to use reported by first responders but not by professional drivers. Reasons for not owning wearable devices and cost could be related; however, this is beyond the scope of this study but warrants further investigation to address all the barriers reported.

### 4.6. Limitations and contributions

A possible limitation of this study was that the 2 surveys were administered at different times (2019 for first responders and 2022 for professional drivers) but both surveys were administered via an online Microsoft form and were very similar. A major strength of this study was strong statistical models. Another strength of this study was access to subject matter experts in developing the survey. Education about health indicators for health monitoring was not assessed in this study but it could be addressed in a future study to determine if this might be a factor in preference for health monitoring. Even though others monitoring health data for professional drivers was a strong predictor for using wearable technology, there were more variables that have not been explored yet that might be predictors for using wearable technology.

Overall based on qualitative response data, there was evidence that cost and durability may provide insight on reasons for differences in history of wearable technology use between the 2 occupations. This warrants further investigation for future studies to determine the importance of cost and durability as barriers to use of wearable technology. Another potential factor that was not included in this study was the financial status of the 2 occupational groups, which might provide further insight for differences between the use of wearable technology. Finally, the education level of the participants should be ascertained in future studies as this might limit the willingness to monitor health indicators.

### 4.7. Conclusion

Occupation appeared to be most important after initial analysis with significant differences observed at the occupational level; however, deeper analysis uncovered characteristics of individuals within the occupations that became more salient to the use of wearable technology. Those characteristics were exposure to HAZMAT, someone else monitoring health data, and experience with wearable technology use. From a public health perspective, first responders and professional drivers are part of a high-risk occupational population, and therefore may benefit from health monitoring. Other potential characteristics should be explored regarding perceptions and attitudes toward health monitoring, which might explain potential barriers as well. This information can be useful to identify types of sensors required for the highly rated health indicators identified to be important in this study, which can be integrated with the dashboard application system. Wearable technology should be considered as a tool for monitoring health and safety of any occupational groups that are at greater risk for occupational exposures, especially related to HAZMAT.

## Author contributions

A.F. conceived the idea and guided the manuscript development; A.Y. along with graduate students (Troy Suwondo and Jacob Grothe) created the survey and collected the data; C.B. verified and analyzed the data; S.T. and S.J. wrote the original manuscript. All authors contributed to revising the manuscript and approved the final version manuscript.

## Funding

This research was funded by the US Department of Transportation, Mid-America Transportation Center grant number 69A355174107. This content reflects solely the opinions of the authors and does not necessarily represent the official views of the US Department of Transportation.

## Conflicts of interest

The authors declare that they have no conflicts of interest.

## Data availability

Data are available from the corresponding author upon reasonable request.

## References

[1] Horan KA , Marks M, Ruiz JM, Bowers CA, Cunningham A. Here for my peer: the future of first responder mental health. Int J Environ Res Public Health. 2021;18(21):11097. 10.3390/ijerph182111097 34769617 PMC8582745

[2] Yung M , Du B, Gruber J, Hackney AA, Yazdani A. Fatigue measures and risk assessment tools for first responder fatigue risk management: a scoping review with considerations of the multidimensionality of fatigue. Saf Sci. 2022;154:105839. 10.1016/j.ssci.2022.105839

[3] Emergency Preparedness and Response: Occupational Safety and Health Risks. NIOSH/CDC; 2018. https://www.cdc.gov/niosh/programs/epr/risks.html

[4] International Association of Firefighters. *Carbon Monoxide Poisoning and Fire Fighters*. 2020. https://www.iaff.org/carbon-monoxide/

[5] Kim YT , Kim WJ, Choi JE, et al. Cohort profile: firefighter research on the enhancement of safety and health (FRESH), a prospective cohort study on Korean firefighters. Yonsei Med J. 2020;61(1):103-109. 10.3349/ymj.2020.61.1.103 31887807 PMC6938775

[6] Barros B , Oliveira M, Morais S. Firefighters’ occupational exposure: contribution from biomarkers of effect to assess health risks. Environ Int. 2021;156:106704. 10.1016/j.envint.2021.106704 34161906

[7] Chen GX , Amandus HE, Wu N. Occupational fatalities among driver/sales workers and truck drivers in the United States, 2003-2008. Am J Ind Med. 2014;57(7):800-809. 10.1002/ajim.22320 24811905 PMC4565185

[8] Joseph L , Standen M, Paungmali A, Kuisma R, Sitilertpisan P, Pirunsan U. Prevalence of musculoskeletal pain among professional drivers: a systematic review. J Occup Health. 2020;62(1):e12150. 10.1002/1348-9585.12150 32810918 PMC7434558

[9] Guest A , Chen Y, Pearson N, King JA, Paine NJ, Clemes SA. Cardiometabolic risk factors and mental health status among truck drivers: a systematic review. BMJ Open. 2020;10(10):e038993. 10.1136/bmjopen-2020-038993 PMC7590350 33099498

[10] Tremblay M , Albert WJ, Lavallière M, et al. Occupational health profile of Canadian Maritimes truck drivers. Work. 2020;67(1):251-257. 10.3233/wor-203270 32955486

[11] Uddin M , Huynh N. Factors influencing injury severity of crashes involving HAZMAT trucks. Int J Transp Sci Technol. 2018;7(1):1-9. 10.1016/j.ijtst.2017.06.004

[12] Chen F , Chen S. Injury severities of truck drivers in single- and multi-vehicle accidents on rural highways. Accid Anal Prev. 2011;43(5):1677-1688. 10.1016/j.aap.2011.03.026 21658494

[13] Cori JM , Downey LA, Sletten TL, et al. The impact of 7-hour and 11-hour rest breaks between shifts on heavy vehicle truck drivers’ sleep, alertness and naturalistic driving performance. Accid Anal Prev. 2021;159;106224. 10.1016/j.aap.2021.106224 34192654

[14] Craft R. Crashes Involving Trucks Carrying Hazardous Materials *.* Federal Motor Carrier Safety Administration; 2004. Accessed November 29, 2023. https://rosap.ntl.bts.gov/view/dot/55

[15] Robinson CF , Burnett CA. Truck drivers and heart disease in the United States, 1979-1990. Am J Ind Med. 2005;47(2):113-119. 10.1002/ajim.20126 15662648

[16] Jalilian H , Ziaei M, Weiderpass E, Rueegg CS, Khosravi Y, Kjaerheim K. Cancer incidence and mortality among firefighters. Int J Cancer. 2019;145(10):2639-2646. 10.1002/ijc.32199 30737784

[17] Pillutla P , Li D, Ahmadi N, Budoff MJ. Comparison of coronary calcium in firefighters with abnormal stress test findings and in asymptomatic nonfirefighters with abnormal stress test findings. Am J Cardiol. 2012;109(4):511-514. 10.1016/j.amjcard.2011.09.044 22105785

[18] Melnikova N , Wu J, Yang A, Orr M. Acute chemical incidents with injured first responders, 2002-2012. Disaster Med Public Health Prep. 2018;12(2):211-221. 10.1017/dmp.2017.50 28760164 PMC5794641

[19] Burgess JL , Nanson CJ, Bolstad-Johnson DM, et al. Adverse respiratory effects following overhaul in firefighters. J Occup Environ Med. 2001;43(5):467-473. 10.1097/00043764-200105000-00007 11382182

[20] Perroni F , Guidetti L, Cignitti L, Baldari C. Psychophysiological responses of firefighters to emergencies: a review. Open Sports Sci J. 2014;7(1):8-15. 10.2174/1875399x01407010008

[21] Baur DM , Christophi CA, Tsismenakis AJ, Cook EF, Kales SN. Cardiorespiratory fitness predicts cardiovascular risk profiles in career firefighters. J Occup Med. 2011;53(10):1155-1160. 10.1097/jom.0b013e31822c9e47 21915073

[22] Dobson M , Choi B, Schnall PL, et al. Exploring occupational and health behavioral causes of firefighter obesity: a qualitative study. Am J Ind Med. 2013;56(7):776-790. 10.1002/ajim.22151 23335437

[23] Poston WSC , Haddock CK, Jahnke SA, Jitnarin N, Tuley BC, Kales SN. The prevalence of overweight, obesity, and substandard fitness in a population-based firefighter cohort. J Occup Environ Med. 2011;53(3):266-273. 10.1097/jom.0b013e31820af362 21386691 PMC5826653

[24] Benstowe SJ . Long Driving Hours and Health of Truck Drivers*.* MS thesis. New Jersey Institute of Technology; 2008. https://digitalcommons.njit.edu/theses/321

[25] Birdsey J , Sieber WK, Chen GX, et al. National survey of US long-haul truck driver health and injury. J Occup Environ Med. 2015;57(2):210-216. 10.1097/jom.0000000000000338 25654523

[26] Dias D , Cunha JPS. Wearable health devices—vital sign monitoring. Sensors (Basel). 2018;18(8):2414. 10.3390/s18082414 30044415 PMC6111409

[27] Greenfield R , Busink E, Wong CP, et al. Truck drivers’ perceptions on wearable devices and health promotion: a qualitative study. BMC Public Health. 2016;16(1):677. 10.1186/s12889-016-3323-3 27475984 PMC4967500

[28] Ribeiro DMD , Colunas MFM, Marques FAF, Fernandes JM, Cunha JPS. A real time, wearable ECG and continuous blood pressure monitoring system for first responders. Paper presented at: 2011Annual International Conference of the IEEE Engineering in Medicine and Biology Society; 2011; Boston, MA. 10.1109/iembs.2011.6091736 22255923

[29] O'Flynn B , Brahmi I, Oudenhoven J et al. First responders occupancy, activity and vital signs monitoring – SAFESENS. Int J Adv Networks Services. 2018; 11(1-2):22‐32. http://www.iariajournals.org/networks_and_services/netser_v11_n12_2018_paged.pdf.

[30] Arachchige SNKK , Burch RF, Chander H, Turner AJ, Knight AC. The use of wearable devices in cognitive fatigue: current trends and future intentions. Theor Issues Ergon Sci. 2021;23(3):374-386. 10.1080/1463922x.2021.1965670

[31] Bergmann J , Chandaria V, McGregor A. Wearable and implantable sensors: the patient’s perspective. Sensors. 2012;12(12):16695-16709. 10.3390/s121216695 23443394 PMC3571806

[32] Patel VM , Chesmore A, Legner C, Pandey S. Trends in workplace wearable technologies and connected-worker solutions for next-generation occupational safety, health, and productivity. Adv Intell Syst. 2022;4(1):2100099. 10.1002/aisy.202100099

[33] Ishwarya G , Rajkumar D. Analysis of ergonomic risk factors in construction industry. Materials Today: Proc. 2021;37:2415-2418. 10.1016/j.matpr.2020.08.269

[34] Koydemir HC , Ozcan A. Wearable and implantable sensors for biomedical applications. Annu Rev Anal Chem. 2018;11(1):127-146. 10.1146/annurev-anchem-061417-125956 29490190

[35] Binkley PF , Frontera W, Standaert DG, Stein J. Predicting the potential of wearable technology. IEEE Engineering in Medicine and Biology 2003;22(3):23-27. 10.1109/memb.2003.1213623

[36] Medcalf S , Hale ML, Achutan C, Yoder AM, Fruhling A, Shearer SW. Requirements gathering through focus groups for a real time emergency communication system for HAZMAT incidents (REACH). J Pub Health Issue Pract. 2021; 5(2):188. 10.33790/jphip1100188.

[37] Grothe J , Tucker S, Blake A, et al. Exploring first responders’ use and perceptions on continuous health and environmental monitoring. Int J Environ Res Public Health. 2023;20(6):4787. 10.3390/ijerph20064787 36981694 PMC10048923

[38] Suwondo T . The perception of technology on continuous health and environmental monitoring for professional drivers. Capstone Experience. 2022; 203. https://digitalcommons.unmc.edu/coph_slce/203.

[39] Choi B , Hwang S, Lee S. What drives construction workers' acceptance of wearable technologies in the workplace?: indoor localization and wearable health devices for occupational safety and health. Autom Constr. 2017;84:31-41. 10.1016/j.autcon.2017.08.005

[40] Häikiö J , Kallio J, Mäkelä S-M, Keränen J. IoT-based safety monitoring from the perspective of construction site workers. Int J Occup Environ Saf. 2020; 4(1):1‐14. 10.24840/2184-0954_004.001_0001.

